# The biomarkers of immune dysregulation and inflammation response in Parkinson disease

**DOI:** 10.1186/s40035-016-0063-3

**Published:** 2016-08-26

**Authors:** Li Chen, Mingshu Mo, Guangning Li, Luan Cen, Lei Wei, Yousheng Xiao, Xiang Chen, Shaomin Li, Xinling Yang, Shaogang Qu, Pingyi Xu

**Affiliations:** 1 Department of Neurology, The First Affiliated Hospital of Guangzhou Medical University, Guangdong, 510120 China; 2 Department of Neurology, The First Affiliated Hospital of Sun Yat-sen University, Guangdong, 510080 China; 3 Department of Neurology, The Third Affiliated Hospital of Sun Yat-sen University, Guangdong, 510082 China; 4 Ann Romney Center for Neurologic Disease, Brigham and Women’s Hospital, Harvard Medical School, Boston, MA 02115 USA; 5 Department of Neurology, The Third Affiliated Hospital of Xinjiang Medical University, Urumqi, 830011 China; 6 Department of Blood Transfusion, The Fifth Affiliated Hospital, Southern Medical University, Guangzhou, Guangdong 510900 China; 7 Department of Neurology, The Affiliated Huadu Hospital of Southern Medical University, Guangzhou, 510800 China

**Keywords:** Parkinson’s disease, α-synucleinopathy, Inflammation, Biomarkers

## Abstract

Parkinson’s disease (PD) is referring to the multi-systemic α-synucleinopathy with Lewy bodies deposited in midbrain. In ageing, the environmental and genetic factors work together and overactive major histocompatibility complex pathway to regulate immune reactions in central nerve system which resulting in neural degeneration, especially in dopaminergic neurons. As a series of biomarkers, the human leukocyte antigen genes with its related proteomics play cortical roles on the antigen presentation of major histocompatibility complex molecules to stimulate the differentiation of T lymphocytes and i-proteasome activities under their immune response to the PD-related environmental alteration and genetic variation. Furthermore, dopaminergic drugs change the biological characteristic of T lymphatic cells, affect the α-synuclein presentation pathway, and inhibit T lymphatic cells to release cytotoxicity in PD development. Taking together, the serum inflammatory factors and blood T cells are involved in the immune dysregulation of PD and inspected as the potential clinic biomarkers for PD prediction.

## Background

The raised incidence of Parkinson’s disease (PD) becomes a serious issue in an aged society [1]. It is known that PD patients in Asia is nearby 11.3 % of movement disorders, slightly lower than that in north America which is up to 13.6 % and 16.6 % of Europe [1]. Based on the characteristic protein conformation and function, central nervous degenerative diseases involved in movement disorders can be clinically classed as α-synucleinopathy, Tauopathy and TDP-43 proteinopathy in which PD belongs to α-synucleinopathy [2]. According to the latest clinic diagnosis criteria of MDS (Movement Disorder Association 2015), the diagnosis of PD need to meet the following criteria: rest tremor and bradykinesia in limbs, clinic symptoms of PD are effectively improved by L-dopa, disease duration is usually accompanied by non-motor symptoms especially in early stage, and discrimination from other neural disease [3]. The various types of α-synuclein manifest mainly as monomers, oligomers, ribbons and fibrils, and a-synuclein diffuses through blood brain barrier, spread around whole nervous system, even invade other tissues or organs such as nasal mucosa, skin, kidney, liver, heart, intestinal tract and salivary glands [4, 5]. As well-known, aging is confirmed to be an important risk to PD [6]. Other risk factors include harmful environment factors and deficient genetic factors [6]. It's worth noting that environment factors such as MPTP, LPS, rotenone or other organic chemicals not only impair dopaminergic (DA) neurons directly and render the epigenetic variation of DNA methylation, but also activate the secondary inflammation/immunity reaction and significantly increase the PD morbidity [7, 8].

## Immunologic dysregulation in PD

Recently immunologic systems were found in central neural system (CNS) which involved with PD [9]. The classic concepts pointed out that central immune system is not directly connected with extracranial system. However, latest reports demonstrated that lymphatic ducts hind in the meninge and link to deep cervicalvenous system in which abundant T, B lymphocytes and dendritic cells are response to environment and genetic variation [10]. According to recent dynamic experiments, the lymphatic system plays an important role in the drainage of spinal fluid to lymphatic duct oriented into deep cervical venous system [10]. This discovery revealed that there is a close linkage between the meninge lymphatic system and α-synuclein transmission. The brain autopsy from PD patients and PD animal models showed that abnormal accumulation of microglia were activated in brain tissues, especially in nigrostriatal area [11]. Under the abnormal inflammation condition, macrophages pathologically diffuse from blood vessels to CNS and transform into microglias contributed to the pathological development of intracranial neural diseases [12]. Meanwhile the activated T lymphocytes were demonstrated to move out from central lymph ducts to attack central neurons, neural myelination or fibers, which resulting in multiple neuropathy as multiple sclerosis like symptoms [13]. In 6-hydroxydopamine-PD rats, there were abundant CD^3+^, CD^4+^, CD^8+^ T lymph cells migrated from blood vessels into substantia nigra (SN) to attack DA neurons [13, 14]. Therefore, it is reasonable to suppose these lymph cells could be the potential biomarkers for the evaluation of PD progression [14].

## Immunoproteasome system in PD

The α-synuclein aggregation is a critical ignition role for the immune disorder in PD [15]. In aging process, the progressive dysfunction of T, B lymph cells and macrophages were related with immune elimination which gradually gave rise to α-synuclein aggregation and neurons degeneration [16, 17]. Furthermore, due to the decreased activity of proteasome, its components as β1,β2 and β5 subunits which compose of 20S proteasome will be replaced by homogenous β1i (large multi-functional protease 2, LMP2), β2i (multi catalytic endopeptidase complex-like-1, MECL-1) and β5i (large multi-functional protease 7, LMP7) subunits and develop a new immunoproteasome system named as i-proteasome [18]. The crystal structure showed that i-proteasomes have more enzyme domains, stronger enzymatic activity, and more effective capacity to degrade the α-synuclein proteins. Particularly, i-proteasome presents the degraded peptides as antigens to special T cells [19]. These antigens combined with major histocompatibility complex molecules (MHC) to ignite another pattern of protein clearance or abnormal neural inflammation [19]. It was reported that i-proteasome was up-regulated in senile hippocampus which involves in the clearance of accumulated amyloid precursor protein (APP) in Alzheimer's disease (AD) [20]. In epilepsy, the i-proteasomes activated by oxidative damaged proteins can maintain the stability of protein metabolism in brain [21]. There are seven i-proteasome cleavage sites in the amino acid sequence of α-synuclein (sequence No.100, 8, 10, 55, 125, 45 and 18, respectively) [22]. Among them, No. 10 and 45 amino acid locate at N terminal named as KTKEGV sequence [22]. This KTKEGV sequence is vital to keep the metabolic stability of α-synuclein [22]. The i-proteasomes mainly guide the MHC molecules to mediate inflammation/immunity reaction, however we did not completely understand the detail about i-proteasomes participating in PD pathogenesis. It is known that after the α-synuclein degraded into 5–15 amino acid residues by ubiquitin proteasome system (UPS), the short antigenic peptides will be transported into endoplasmic reticulum system by the transporter associated with antigen processing (TAP) which brought the antigenic peptides to cell surfaces and the complex will be recognized by the MHC-I and MHC-II receptors. Then MHC presentation activates CD^4+^T cells and CD^8+^cytotoxic T lymphocytes (CTL) to take part in the immune regulation in CNS, respectively [23]. Human MHC molecules are also called human leukocyte antigen (HLA). DA neurons express multiple HLA molecules and present the digested peptides on the surface recognized by CD^8+^ CTL cells [23]. At the same time, the activated microglias secrete TNF-α, IL-1β and IL-6 inflammatory factors to attack DA neurons, or present antigens to CD^4+^ T cells by MHC-II pathway [24, 25]. Abundant MHC-II positive microglia and CD^4+^,CD^8+^ T cells were found in the SN of PD patients, and the microglia activity was confirmed to be related to the degeneration severity of DA neurons as well as PD progression [17]. Our data found that natural killer (NK) cells increased and CD^3+^T, CD^3+^CD^4+^T cells, Th1 cells decreased in the peripheral blood from PD patients [26]. These immune reactions will result in the increased secretion of TNF-α, IL-2, IL-6, IL-10, IL-1β and IFN-γ in cerebrospinal fluid (CSF) or blood, and accompanied with more than ten kinds of high sensitive and specific antibodies increased, such as elongation factor 1-alpha1 and poly (A) binding protein cytoplasmic-3 [25, 27]. The surface antigens in monocytes were up-regulated and correlated with the Unified Parkinson's Disease Rating Scale (UPDRS) III scores of PD patients, indicating the importance of central and peripheral immunity reaction occurred in PD [28]. Recent studies showed that HLA-I in DA neurons and HLA-II in activated microglia both participate the antigen presentation and α-synuclein degradation [24, 29]. When proteasome function is suppressed by intracellular α-synuclein accumulation, i-proteasome will be activated as compensatory to promote α-synuclein degradation [30]. The degraded peptides will be presented to CD^8+^T cells by MHC-I molecules, and the CD^8+^T cells release pre-inflammatory factors and activate Wnt/Nurr1 and Nuclear factor-kB signal pathways to ignite the secondary inflammation reaction [30, 31]. Meanwhile, extracellular α-synuclein integrates with HLA-DR molecules in microglia, mediate the activation of CD^4+^T cells by MCH-II antigen presentation pathway and T lymphocytes to participate the immune reaction in CNS [15, 25]. If the proteasomes function are impaired in DA neurons, α-synuclein accumulation could activate the i-proteasomeas as compensatory to provide much more MHC-I and MHC-II molecules to CD^8+^T and CD^4+^ T cells showing stronger immune response [29, 32]. Under the condition of HLA gene mutation, the genetic risks may profoundly affect the α-synuclein degradation, result in the immune dysregulation in CNS and finally accelerate the DA neurons apoptosis.

## Immunological genes involved in PD

It is well known that genetic factors has high risk for PD. Much data revealed that the variation of HLA genes severely accelerate PD development [33]. Total 224 genes in HLA region are located at I, II and III region of HLA genomics, respectively [33]. Excepted the rarely expressed genes at HLA-I region, 128 genes from II and III of HLA region were found the contribution to immune regulation in CNS [33]. For example, HLA-DR at HLA II region is composed of DRA and DRB genes which encoding α and β chain respectively [33]. The α and β chains are assembled into HLA-DR molecules contributed to the immunologic response in neuron degeneration [33]. It's worth noting that HLA genes have high polymorphism among different ethnic and geographical population [34]. For example, HLA-C*0304, HLA-DRB1*0404, HLA-DRA and HLA-DQB1 were closely related to PD in European population, but no reports in Japanese [33, 34]. Our investigation firstly showed that HLA-DRB1*0301 is a suspect locus to PD in Chinese Han population (OR = 2.048,95%CI:1.455,2.884), but no meaningful polymorphism found in Chinese Uyghur population [34]. It is known that proteasome subunit beta (PSMB) genes locate at HLA-II region and adjacent to HLA-DR genes [33]. PSMB genes encodes β chain to help the α-synuclein degradation after the proteasome constitution [35]. It was speculated that PSMB may cooperate with HLA-DR to participate the immune regulation and intrinsic antigen presentation [36]. Our investigation has confirmed that female carrying rs17587 G/G at PSMB9 will significantly increase PD risk (OR = 1.851 95%CI: 1.240). The rs17587 locates at the code region of exon 60 at PSMB9 [33]. Its variation of nucleotide G to A may lead to the amino acid of arginine replaced with histidine [37]. The G/G genotype of rs17587 at PSMB9 disrupts the peptide cleavage site of proteasome resulting in the impaired activity of proteasome [37]. Other important genes are reported to involve in immunological regulation of PD, included HLA, GBA (Glucosidase Beta Acid), SNCA (Synuclein alpha), LRRK2 (Leucine-rich repeat kinase 2) and NURR1 (Nuclear receptor related 1 protein) [33, 38]. The GBA protein is liable to regulate the abnormal activation of microglia in basal ganglia [39], and the LRRK2 protein may affect the inflammatory reaction medicated by Toll-like receptor 4 [40]. Nurr1, a new anti-inflammatory factor, regulate the nuclear factor-kB-p65 signaling pathway in microglia and astrocytes through Nurr1/CoREST (RE1-silencing transcription factor co-repressor 1) complex [41]. Generally, variations in these PD-related genes may promote the antigen presentation and the activation of T and B lymphocytes, strengthen the sensitivity of DA neurons to environmental risk, and down-regulate the catecholamine metabolism conducted by MHC pathway [34]. In other words, it was suggested these genetic factors, serum immune/inflammatory molecules and blood lymphocytes may be the potential clinic biomarkers for PD prediction.

## Dopamine receptors in blood lymphocytes

Dopamine receptors were found in the blood white cells of human kind [42]. These receptors are moderately expressed in neutrophil granulocyte or eosinophil granulocyte, but highly expressed in B lymphocytes and NK cells [42]. There are five kinds of dopamine receptors in CD^3+^CD^4+^T lymphocytes with different expression level [42]. For example, the D1 receptor expression is significantly higher than D2, and D5 receptor expression is higher than D1 in T lymphocytes and memory T lymphocytes [42]. The D2 receptor showed no significant different expression in blood cells from PD patient [42, 43]. But the D3 receptor expression declines dramatically in peripheral lymphocytes and is associated with PD severity [42]. The proportion of CD95/CD3 increases significantly in the lymphocytes from PD patients, but decreases significantly after L-dopa treatment [44]. The activation of dopamine receptors by different activators can reduce the release of TNF-α, IL-6 and other cytokines from microglia which may help to explain their neuro-protection [45, 46].

## MicroRNA signatures in PD

Recent studies demonstrated that some PD-related genes is modulated by 18 ~ 25 nt microRNAs (miRNAs) [47] at post-transcriptional level. Matching with target mRNA 3′ transcription terminals, miRNAs guide Dicer enzyme to degrade the mRNA targets or restrain their translation [48]. There are multiple mechanisms of miRNAs regulation on PD pathogenesis. For example, miR-7 and miR-153 modulate the mRNA level of α-synuclein [49], LRRK2 contributes to the regulation of E2F1/DP expression which is affected by miR-7 and miR-184 [50]. Recent studies mainly focused on the microglia activation and macrophage infiltration in brain. It becomes a hot spot about the mechanism of miRNAs modulating the related inflammatory disorders in CNS. Based on function classification, some microRNAs are classified as pro-inflammatory molecules, such as miR-155, miR-125 and miR-101, and some are anti-inflammatory molecules, such as miR-146, miR-21 and miR-124 [51]. It was reported that miR-146 modulates the microglia activation in CNS regulated by NF-KB and JNK-STAT (Janus kinase/signal transducers and activators of transcription) pathways, miR-124 retrains macrophage and microglia activation conducted by CCAAT-enhancer-binding proteins-α–PU.1 complex [52, 53]. All the miRNAs are involved in the inflammation/immunity system on PD pathogenesis [54]. Furthermore, the decreased level of miR-141, miR-214、miR-146b-5p and miR-193a-3p was found closely associated with the early stage of PD [55]. In our study, there were 644 abnormal miRNAs detected in A53T-PD transgenic mice using high-throughput screening. Among them, 72 miRNAs were confirmed to involve in the immune response in SN. The level of 42 miRNAs has changed significantly in midbrain, such as miR-146b-5 decreases to 0.45 fold, whereas miR-124-5p up to 0.44 fold. Several miRNAs were confirmed to be stable in blood, urine and CSF, so called circulating miRNAs [56]. We also screened out 10 circulating miRNAs in CSF samples from 40 Chinese PD patients for disease’s inflammatory evaluation [57]. Among them, miR-200a-3p and miR-542-5p were supposed to be closely related to α-synuclein aggregation, whereas miR-342-5p was proved to participate in modulating inflammatory reaction [57]. These circulating miRNAs are ideal biomarkers for the evaluation of CNS diseases.

## Conclusions

In summary, the interaction of aging, environment risk and genetic factors results in the accumulation of α-synuclein in midbrain which ignites the special inflammatory /immune response medicated by i-proteasome. In the inflammatory/immune procession, MHC signal pathway accompanied with microglia activation is vital to DA neurons apoptosis. Taking together, α-synuclein accumulation and abnormal MHC antigenic presentation cause inflammation/immune response in CNS and provide specific biomarkers for the prediction of DA neurons degeneration and assessment of PD risk as well as PD development (Table 1).

**Table 1:** The biomarkers of immune dysregulation and inflammation response in PD

Immunological genes	PSMB9-rs17587, HLA-DRA, HLA-DRB1*0404, HLA-DRB1*0301, HLA-C*0304, HLA-DQB1, etc.
PD related genes	PARK1, PARK2, PINK1, DJ-1, ATP13a2, GBA, RAB7L1-NUCKS1, STK39, BST1, FAM47E-SCARB2, SNCA, HLA, GPNMB, FGF20, LRRK2, GCH1, MAPT, SREBF1, DDRGK1, NURR1, etc.
Proteins in CSF	a-synuclein, Aβ42, Tau, Nurr1, etc.
Inflammation factors	TNF-α, IL-2, IL-6, IL-10, IL-1β, IFN-γ, etc.
Antibodies	ICAM4, PTCD2, FRMD8, CTLA-4/Fc, MYOT, HSH2D, FN1, TRIM21, Elongation factor 1-alpha 1, PABPC3, etc.
Blood lymphocytes	CD^3+^, CD^4+^, CD^8+^, CD^31+^ α4β^7+^ CD^4+^, CD95/CD3, NK cells, etc.
PD related miRNAs	miR-7, miR-153, miR-155, miR-125, miR-101, miR-146, miR-21, miR-124, miR-141, miR-214, miR-146b-5p, miR-193a-3p, miR-200a-3p, miR-542-5p, miR-342-5p, etc.

## Abbreviations

AD: Alzheimer's disease
APP: Amyloid precursor protein
CNS: Central neural system
CoREST: RE1-silencing transcription factor co-repressor 1
CSF: Cerebrospinal fluid
CTL: Cytotoxic T lymphocytes
DA: Dopaminergic
GBA: Glucosidase beta acid
HLA: Human leukocyte antigen
JNK-STAT: Janus kinase/signal transducers and activators of transcription
LMP2: Large multifunctional protease 2
LMP7: Large multifunctional protease 7
LRRK2: Leucine-rich repeat kinase 2
MDS: Movement Disorder Association
MECL-1: Multicatalytic endopeptidase complex-like-1
MHC: Major histocompatibility complex molecules
miRNAs: microRNAs
NK: Natural killer
NURR1: Nuclear receptor related 1 protein
PD: Parkinson’s disease
PSMB: Proteasome subunit beta
SN: Substantia nigra
SNCA: Synuclein alpha
TAP: Antigen processing
UPDRS: Unified Parkinson’s Disease Rating Scale
UPS: Ubiquitin proteasome system
